# The association and clinicopathological significance of Integrin alphavbeta6 and Rac1 expression in gastric carcinoma

**DOI:** 10.3389/fonc.2024.1347270

**Published:** 2024-01-24

**Authors:** Junjian Yu, Wenyu Jia, Qi Liu, Antai Yuan, Zhuoyu Jia, YuQi Sun, Zequn Li, Shougen Cao

**Affiliations:** ^1^ Department of Gastrointestinal Surgery, The Affiliated Hospital of Qingdao University, Qingdao, Shandong, China; ^2^ Department of Endocrinology, Qingdao Municipal Hospital, Qingdao, Shandong, China

**Keywords:** ITGB6, Rac1, gastric cancer, immunohistochemistry, prognosis

## Abstract

**Background:**

The present study investigate the expression and correlation of ITGB6 and Rac1 proteins in gastric cancer tissues. By exploring the clinical significance and functions of these proteins, we aimed to gain further insights into the mechanisms underlying gastric cancer development.

**Patients and methods:**

In this study, a total of 198 patients diagnosed with gastric cancer and who underwent gastrectomy between July 2010 to October 2012 were included. The median follow-up time was 52.00 months. To evaluate the factors influencing overall survival, Kaplan-Meier survival curve analysis and Cox regression analysis were conducted. Furthermore, an independent prognostic factor-based nomogram was constructed and validated to predict survival outcomes in gastric cancer patients. In addition, *in vitro* experiments including CCK8 and Transwell assays were conducted to explore the roles of ITGB6 and Rac1 in gastric cancer.

**Results:**

The expression levels of ITGB6 and Rac1 in gastric cancerous and paraneoplastic tissues were detected by immunohistochemistry. The correlation and clinical significance of the two proteins were also investigated. ITGB6 expression showed significant associations with tumor size (*P*=0.030), pathological grading (*P*=0.013), location (*P*=0.031), N stage (*P*=0.002), and clinical stage (*P*=0.002). Additionally, we found that tumor size (*P*=0.013), tumor’s anatomical location (*P*=0.031), N stage (*P*=0.002), clinical stage (*P*=0.035), and survival status (*P*<0.001) were significantly associated with the expression of Rac1. ITGB6 was moderately correlated with Rac1 (*r*=0.285, *P*<0.001). Both the Kaplan-Meier survival analysis and Cox regression model analysis demonstrated that the presence of positive expression of ITGB6 and Rac1 proteins served as independent prognostic factors for gastric cancer. These findings highlight the potential of ITGB6 and Rac1 as valuable markers for predicting the prognosis of gastric cancer patients (HR=2.212 *P*<0.001 and HR=2.073 *P*=0.001), with a significant poorer trend for 5-year survival (*P*<0.0001, respectively, the log-rank test). Additionally, subsequent *in vitro* experiments preliminarily demonstrated that ITGB6 and Rac1 promoted the proliferation, migration and invasion of gastric cancer cells, and ITGB6 may functions via targeting Rac1.

**Conclusion:**

ITGB6 and Rac1 are indicators of poor prognosis and tumor progression in gastric cancer patients. The potential signaling pathways associated with both may provide useful targets for the prevention and treatment of gastric cancer.

## 1 Introduction

Gastric cancer is indeed a significant global health concern that warrants attention. It ranks as the fifth most commonly diagnosed cancer worldwide and is the third leading cause of cancer-related deaths. The prevalence of this disease underscores the importance of ongoing research, early detection strategies, and effective treatment options to combat gastric cancer and improve patient outcomes (1, 2). Indeed, integrin alphavbeta6 (ITGB6) and Rac1 have been recognized as playing crucial roles in the occurrence and progression of gastric cancer (3–5).

ITGB6 is classified as a cell surface receptor and is a member of the integrin protein family, consisting of αv subunit and β6 subunit that form a heterodimeric structure. Integrins function as transmembrane receptors, facilitating cellular interactions with both neighboring cells and the extracellular matrix (ECM). They play critical roles in crucial cellular processes such as adhesion, migration, and signaling transduction. It is widely expressed in various of cell types, including epithelial cells, fibroblasts, and endothelial cells (6). In the context of cancer, ITGB6 has been demonstrated to facilitate tumor progression and metastasis by regulating the signaling pathway of transforming growth factor-beta (TGF-beta) and promoting epithelial-mesenchymal transition (EMT), a cellular process in which epithelial cells undergo a transformation into a mesenchymal phenotype, promoting increased cellular motility and invasiveness (7, 8).

Rac1 is a member of the Rho family of small GTPase proteins. It plays a crucial role in governing a range of cellular processes, such as cell proliferation, migration, adhesion, and cytoskeletal organization. Rac1 is activated by a variety of extracellular signals, such as growth factors and cytokines, and it mediates downstream signaling pathways that regulate cell behavior (9). The expression and activation of Rac1 are commonly increased in diverse cancer types, including breast cancer, colon cancer, and lung cancer (10). Furthermore, Rac1 has also been demonstrated to play a role in promoting the occurrence of the EMT (11–13). Due to its significant involvement in oncogenesis and the advancement of cancer, Rac1 has garnered attention as a promising target for therapeutic intervention in cancer management.

Studies have shown that ITGB6 can activate Rac1 signaling in epithelial cells, leading to changes in cell behavior that are associated with cancer progression. For example, ITGB6 has been shown to promote cancer cell invasion by activating Rac1 signaling and promoting the formation of cellular protrusions and cell movement (14). Additionally, ITGB6 has been shown to promote the acquisition of stem-like properties in cancer cells, allowing them to resist chemotherapy and evade immune surveillance, and this is also mediated through Rac1 signaling (15). However, the prognostic implications of ITGB6 and Rac1 overexpression in gastric cancer patients, as well as their potential interrelationship, remain indeterminate. The objective of this study is to explore the association and clinical relevance of Rac1 and ITGB6 expression in gastric cancer.

## 2 Materials and methods

### 2.1 Patients

This study strictly adheres to the STROBE Statement. Our investigation entails a retrospective analysis of individuals who underwent gastrectomy at the Affiliated Hospital of Qingdao University from July 2010 to October 2012. The study population included gastric cancer patients who underwent surgical resection as their initial treatment, experienced no significant perioperative complications, and had complete clinicopathological data and adequate tissue files. We collected tumor tissues and paired adjacent tissues from 198 patients (132 males and 66 females), aged 25-90 years, with a median age of 68 years. Follow-up for all patients ended in April 2022, and the median follow-up duration was 52.00 months.

### 2.2 Data collection

We collected pertinent information regarding demographic and clinical parameters from the study participants, encompassing age, gender, time of operation, pathological type, pathological grading, tumor size, tumor site, lymphatic invasion, lymph node positivity, and the pathological staging of tumors was conducted following the 8th edition of the American Joint Committee on Cancer (AJCC) criteria, which classifies stages I-IV based on tumor-node-metastasis (TNM) characteristics. Overall survival (OS) was defined as the interval starting from the initial surgical resection to either 60 months or the occurrence of patient demise. Resected tumor biopsy samples were subjected to fixation in 10% formalin at 4°C for 24 hours, followed by embedding in paraffin for subsequent histological and immunohistochemical examination. This study followed the principles outlined in the Declaration of Helsinki and was granted approval by the Ethics Committee of the Affiliated Hospital of Qingdao University (No.QDFY27398). Prior to participation, written informed consent was obtained from every individual patient.

### 2.3 Immunohistochemistry and immunoreactivity score

Immunohistochemistry (IHC) was executed utilizing the streptavidin-biotin technique (Universal DakoCytomation LSAB2 system; DAKO, Glostrup, Denmark). Following deparaffinization of the tissue sections, they underwent autoclave heating at 120°C for 5 minutes, followed by antigen retrieval in a citrate buffer solution (2mM citric acid and 9mM sodium citrate dihydrate, pH 6.0). Rabbit polyclonal antibody against ITGB6 (dilution, 1:500; #28378-1-AP; Proteintech; USA) and Rabbit polyclonal antibody against Rac1 (dilution, 1:500; #24072-1-AP; Proteintech; USA) were utilized. The primary antibody was applied to the slides and incubated in a humid chamber at 4°C for 12-18 hours. Detection of bound antibody was achieved using a modified labeled avidin-biotin reagent for 20 minutes, followed by phosphate-buffered saline washing. A 0.1% diaminobenzidine solution served as the chromogen and was applied for 5 minutes. Slides were subsequently counterstained with Mayer’s haematoxylin for 5-10 minutes.

To perform a semiquantitative analysis of the immunoreactivity of ITGB6 and Rac1 receptors, the H-score was utilized in this study (16). More than 500 tumor cells were counted in each case, and the H-score was calculated by summing the percentages of strongly stained nuclei (3×), moderately stained nuclei (2×), and weakly stained nuclei (1×), resulting in a potential range of 0-300. The score was independently determined by two pathologists who identified the immunostained areas on slides using a double-headed light microscope. In this study, the interobserver differences were found to be less than 5%, and the average of the two values was obtained.

### 2.4 Cell culture and transfection

Human gastric carcinoma cell line SCG7901 was obtained from the Cell Bank of the Chinese Academy of Sciences. SCG7901 cells were cultured in RPMI-1640 medium (Gibco, CAT#C11875500BT), supplemented with 10% fetal bovine serum (Gibco, CAT#C11995500BT) and 1% Penicillin/Streptomycin, in a controlled environment with 5% CO_2_ at a temperature of 37°C.

ITGB6-specific small interfering RNAs (siRNAs, Ribobio, Guangzhou, China) were employed to selectively suppress ITGB6 expression in SCG7901 cells. The cells were transfected with siRNA (si-NC; si-ITGB6, 5’-GAAAGAUUGUGUUAGUUAAGU-3’, 5’-UUAACUAACACAAUCUUUCUA-3’) using the transfection reagent Lipofectamine 2000 (Invitrogen). Upon achieving a confluence rate of 70% in the medium, 5 μL of siRNA and Lipofectamine 2000 reagents were gently mixed and the culture was continued for 4 hours within a humid environment containing 5% CO_2_. On the second day subsequent to transfection of SCG7901 cells, a portion of the si-NC and si-ITGB6 cells were incubated in medium supplemented with NSC233766 (50μmol), designated as the si-NC+NSC233766 group and si-ITGB6+NSC233766 group, respectively. NSC23766 (Cat#: 733767-34-5) was obtained from Med Chem Express (NJ, U.S.A.) and dissolved in double distilled water at a concentration of 10 mM for storage purposes. Following an incubation period of at least 48 hours, the cells were utilized for subsequent experiments.

### 2.5 Cell proliferation assay

The Cell Counting Kit-8 (CCK-8, Fude Biological, CAT#FD3788) was employed to assess cellular proliferation. More specifically, cells were seeded onto 96-well plates at an initial density of 2000 cells per well. Following incubation at 37°C for durations of indicated time points, 10 μl of CCK8 solution was added to each well, followed by a coincubation of 2 hours. Subsequently, the absorbance was measured at 450 nm using spectrophotometry.

### 2.6 Transwell invasion/migration assay

The experiment employed a 24 well plate with transwell chamber (Corning Costar, CAT#3422) to conduct cell migration and invasion assays. For the migration assay, 600 μl of RPMI-1640 medium with 10% FBS and 600 μl of serum-free RPMI-1640 medium were added to the upper and lower chambers, respectively. Subsequently, 5×10^4^ cells, with or without an inhibitor, were introduced into the upper chamber. Following a 24-hour incubation period, non-migrated cells were eliminated using cotton swabs. The infiltrated cells were fixed with 4% formaldehyde for 15 minutes and stained with 0.5% crystal violet for 10 minutes at room temperature.

Cell invasion assay followed a similar procedure, with the exception of Matrigel (BD Biosciences, CAT#354277) pre-coating the upper chamber. Microscopic images of the cells were captured and cell counting was performed using both a microscope and ImageJ software. All experiments were repeated three times.

### 2.7 Statistical analysis

The association between ITGB6 and Rac1 expression and clinicopathological variables was evaluated using either the chi-square test or Fisher’s exact test. For categorical variables, frequency and percentage were used. Quantitative data were presented as the mean ± SD. Student’s t test was used to determine the differences between two groups. Differences among multiple groups were determined by one-way analysis of variance (ANOVA). Survival outcomes were examined through the utilization of the Kaplan-Meier method and log-rank test. Using the Receiver Operating Characteristic (ROC) curve, we evaluated the prognostic value of ITGB6 and Rac1 expression in predicting the prognosis and lymph node metastasis in gastric cancer patients. The correlation between ITGB6 and Rac1 expression levels was assessed using the Spearman correlation analysis. Univariate and multivariate analyses of cancer-specific mortality were conducted by implementing the Cox proportional hazards model. Graphics were created using GraphPad Prism 7 (GraphPad Software, Inc, La Jolla, CA, USA) and Photoshop software (Adobe, Version CS5.1). The statistical analysis was carried out by employing the SPSS version 26.0 (IBM, Armonk, NY, USA). In order to establish statistical significance, a significance level of *P*<0.05 was defined.

The nomogram was developed and validated following the established guidelines for constructing nomograms (17, 18). A nomogram was developed using the independent prognostic factors to predict survival outcomes. Furthermore, the nomograms were utilized for prognostic prediction using the RMS package in R software, version 3.1.3 (https://www.r-project.org/). Model performance was assessed through measures of discrimination and calibration (19). The discrimination ability of the nomogram was assessed using Harrell’s concordance index (C-index) (20). The range of the C-index is between 0.5 and 1.0, where a value of 0.5 represents no discrimination, and a value of 1.0 indicates perfect discrimination. A calibration plot was employed to visually assess the congruence between the predicted prognosis from the nomogram and the actual observed prognosis.

## 3 Results

### 3.1 Clinical characteristics

**Table 1** illustrates the clinical and pathological characteristics of 198 patients diagnosed with gastric cancer. Among these individuals, 132 (64.7%) were of the male gender while 66 (32.4%) were female, with an average age of 65.86 ± 11.57 years (ranging from 25 to 90 years). The optimal threshold for age was determined based on the survival duration. Likewise, the optimal thresholds for tumor size were defined in relation to survival time, specifically 1-6.5, 7-9, and 9.5-19, respectively. The critical milestone for the positive lymph node rate was fixed at 42.9% taking into account survival time. The gastric cancer tissues were classified in accordance with the international classification system first proposed by the World Health Organization (WHO) back in 1979. These classifications include adenocarcinoma, mucinous adenocarcinoma, sigma-ring cell carcinoma, and undifferentiated carcinoma. Moreover, the anatomical localization of the tumor served as a basis for categorizing gastric cancer into the upper third (comprising the preventriculus and fundus of the stomach), middle third (corresponding to the body of the stomach), and lower third (including the antrum of the stomach and pylorus), constituting 34.3%, 34.3%, and 31.3%, respectively. The pathologic tumor-node-metastasis (TNM) classification and cancer stage were determined in accordance with the eighth edition of the American Joint Committee on Cancer (AJCC) stage groupings. After undergoing surgery, all patients were diligently monitored, and the median survival period was found to be 52 months (ranging from 0.03 to 71 months).

**Table 1:** The correlation of integrin αvβ6 expression and RAC1 expression with clinicopathologic variables in cases of gastric cancer.

Clinicopathological factors	n	ITGB6 expression		χ2	P Value	RAC1 expression		χ2	P Value
		High(n=79)	Low(n=119)			High(n=72)	Low(n=126)		
Gender Male Female	132 66	52(%) 27(%)	80(%) 39(%)	0.042	0.837	45(%) 27(%)	87(%) 39(%)	0.884	0.347
Age(years) ≤77 >77	165 33	68(%) 11(%)	97(%) 22(%)	0.712	0.399	59(%) 13(%)	106(%) 20(%)	0.157	0.692
Pathological type Adenocarcinoma Adenocarcinoma, mucinous Carcinoma,Signet Ring Cell Carcinoma, Undifferentiated	141 17 38 2	60(%) 5(%) 13(%) 1(%)	81(%) 12(%) 25(%) 1(%)	1.792	0.617	50(%) 6(%) 15(%) 1(%)	91(%) 11(%) 23(%) 1(%)	0.378	0.945
Pathological grading I-II III-IV	57 141	15(%) 64(%)	42(%) 77(%)	6.159	0.013	25(%) 47(%)	32(%) 94(%)	1.944	0.163
The largest diameter 1-6.5 7-9 9.5-19	115 56 27	41(%) 21(%) 17(%)	74(%) 35(%) 10(%)	6.989	0.030	32(%) 27(%) 13(%)	84(%) 29(%) 14(%)	8.641	0.013
location upper third middle third lower third	68 68 62	26(%) 38(%) 18(%)	42(%) 33(%) 44(%)	6.929	0.031	22(%) 33(%) 17(%)	46(%) 35(%) 45(%)	6.965	0.031
Lymphatic invasion No Yes	107 91	37(%) 42(%)	70(%) 49(%)	2.747	0.097	34(%) 38(%)	73(%) 53(%)	2.118	0.146
Nerve invasion No Yes	146 52	56(%) 23(%)	90(%) 29(%)	0.552	0.458	52(%) 20(%)	93(%) 33(%)	0.134	0.714
T stage T1-T2 T3-T4	31 167	8(%) 71(%)	23(%) 96(%)	3.044	0.081	7(%) 65(%)	24(%) 102(%)	3.017	0.082
N stage N0 N1 N2 N3	47 31 52 68	13(%) 6(%) 23(%) 37(%)	34(%) 25(%) 29(%) 31(%)	14.772	0.002	12(%) 9(%) 14(%) 37(%)	35(%) 22(%) 38(%) 31(%)	14.678	0.002
M stage M0 M1	194 4	77(%) 2(%)	117(%) 2(%)	0.174	0.677	69(%) 3(%)	125(%) 1(%)	2.634	0.105
TNM stage I-II III-IV	74 124	19(25.7%) 60(48.4%)	55(74.3%) 64(51.6%)	9.969	0.002	20(27.0%) 52(41.9%)	54(73.0%) 72(58.1%)	4.451	0.035
Survival Death Censored	106 92	59(%) 20(%)	47(%) 72(%)	23.634	<0.001	54(%) 18(%)	52(%) 74(%)	20.956	<0.001
Survival time		26.32±21.42	43.79±21.30			25.94±21.34	43.03±21.56		

The meaning of the red values is P < 0.05.

### 3.2 The manifestation of ITGB6 and Rac1 in conventional and malignant gastric tissue

**Figure 1:**
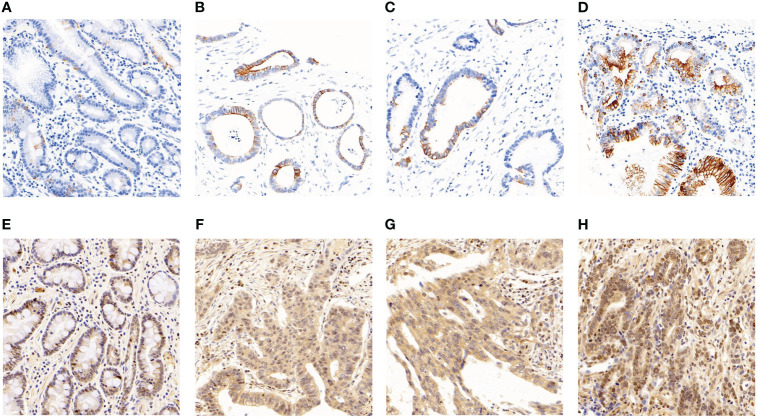
The manifestation of ITGB6 and Rac1 in diverse specimens. **(A)** Immunohistochemical staining exhibited diminished ITGB6 expression in adjacent normal tissues. **(B)** Immunohistochemical staining revealed elevated ITGB6 expression in tumor tissues. **(C)** ITGB6 expression in tumor tissues lacking lymphatic invasion. **(D)** ITGB6 expression in tumor tissues with lymphatic invasion. **(E)** Immunohistochemical staining displayed reduced Rac1 expression in adjacent normal tissues. **(F)** Immunohistochemical staining exhibited heightened Rac1 expression in tumor tissues. **(G)** Rac1 expression in tumor tissues without lymphatic invasion. **(H)** Rac1 expression in tumor tissues with lymphatic invasion.

**Figure 2:**
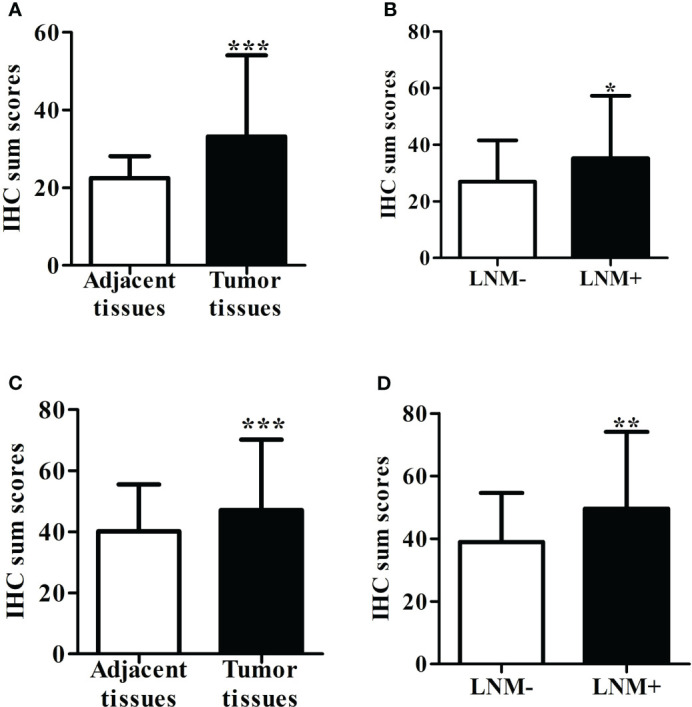
**(A)** The level of ITGB6 expression was assessed using the H-score in both adjacent normal tissue specimens and tumor tissues. **(B)** The H-score of ITGB6 expression was evaluated in adjacent normal tissue specimens, comparing those with lymph node metastasis and those without. **(C)** We measured the H-score of Rac1 expression in both adjacent normal tissue specimens and tumor tissues. **(D)** The H-score of Rac1 expression was analyzed in adjacent normal tissue specimens, comparing those with lymph node metastasis and those without. *P<0.05; **P<0.001; ***P<0.0001.

Immunostaining was conducted to ascertain the expression of ITGB6 and Rac1 in gastric carcinoma specimens in comparison to neighboring normal tissues (**Figures 1A–H**). A semiquantitative H-score, based on the overall staining intensity and extent of positive cells, as previously described, was assigned. By employing the H-score, the expression levels of ITGB6 and Rac1 were determined and compared between gastric carcinoma tissues and adjacent normal tissues within the diagnostic cohort. The results showed that ITGB6 and Rac1 were highly expressed in gastric cancer tissues (H-score, 33.87 ± 22.15, 46.99 ± 24.23, respectively) and lymph node metastases (H-score, 36.73 ± 23.69, 50.29 ± 25.96, respectively) compared with adjacent normal tissues (H-score, 22.52 ± 5.61, *P*<0.001; 40.13 ± 15.37, *P*<0.001, respectively) and non-metastatic tissues (H-score, 25.60 ± 14.19, 37.44 ± 14.91, respectively, **Figures 2A–D**).

### 3.3 Correlation between ITGB6 and clinicopathologic factors and patient prognosis

The X-tile plot was employed to ascertain the most favorable threshold value of the H-score for evaluating the statistical significance pertaining to the overall survival (OS) of patients (H-score=33.2). Classifying the diagnostic cohort based on this threshold, 198 individuals suffering from gastric cancer were partitioned into categories of high and low ITGB6 expression. Among these patients, 119 out of 198 (60.1%) exhibited low levels of ITGB6 expression, while 79 out of 198 (39.9%) showed high levels. The fundamental attributes of these two groups are comprehensively outlined in **Table 1**. ITGB6 expression was significantly associated with pathological grading (*P*=0.013), tumor size (*P*=0.030), location (*P*=0.031), N stage (*P*=0.002), TNM stage (*P*=0.002), positive lymph node rate (*P*=0.002), and survival status (*P*<0.001) (**Table 1**).

**Figure 3:**
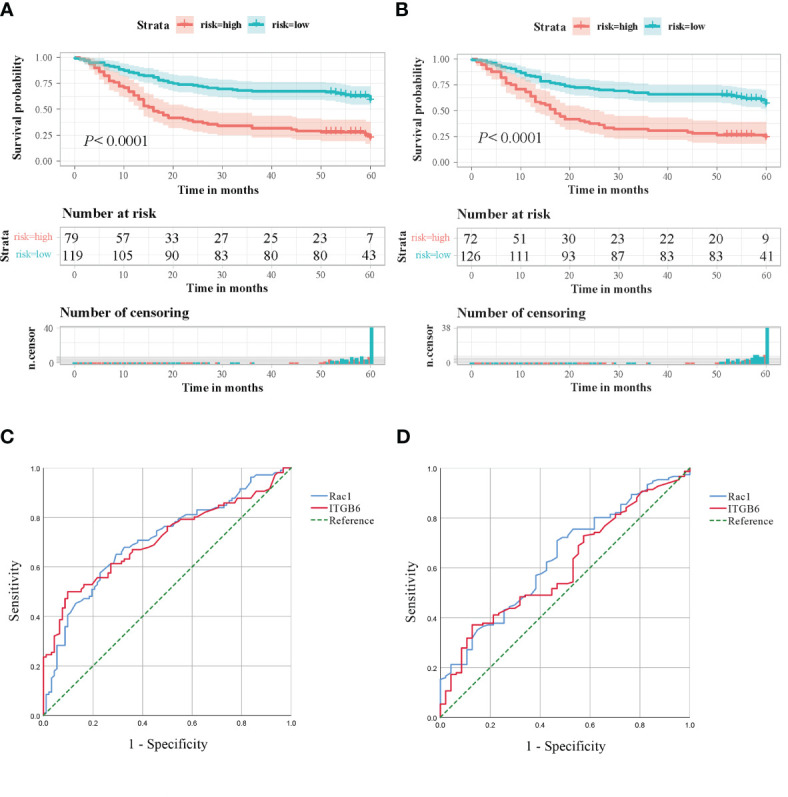
**(A)** Kaplan-Meier survival curves based on the ITGB6 gene expression level. **(B)** Kaplan-Meier survival curves based on the Rac1 gene expression level. **(C)** Receiver Operating Characteristic (ROC) analysis was conducted to predict the prognosis of gastric cancer patients using ITGB6 and Rac1 expression. **(D)** Receiver Operating Characteristic (ROC) analysis was conducted to predict lymph node metastasis in gastric cancer patients using ITGB6 and Rac1 expression.

The examination of Kaplan-Meier survival analysis unveiled that individuals demonstrating elevated ITGB6 expression showcased notably inferior overall survival outcomes in comparison to those with diminished ITGB6 expression(*P*<0.0001; log-rank test, *χ^2^ = *30.845). **Figure 3A** illustrates the correlation between the expression of ITGB6 and the survival rate of patients over time. Subsequently, a time-dependent receiver operating characteristic (ROC) analysis was performed to evaluate the prognostic significance of ITGB6 expression in individuals diagnosed with gastric cancer. This analysis resulted in an area under the curve (AUC) of 0.708, which indicates a moderate predictive value.(95% CI: 0.636-0.780, sensitivity: 52.8%, specificity: 80.4%) (**Figure 3C**). A ROC curve was also constructed to appraise the potential of ITGB6 as a biomarker in prognosticating lymph node metastasis. The area under the curve (AUC) for ITGB6 in discerning patients with lymph node metastasis stood at 0.604 (95%CI:0.516-0.692, sensitivity:41.7%, specificity:76.6%) (**Figure 3D**).

### 3.4 Correlation between Rac1 expression and clinicopathological variables and patient prognosis

To assess the statistical significance of H-score, the X-tile plot was employed to ascertain the optimal threshold value, derived from its association with the overall survival (OS) of patients, which was identified as 47.1. Utilizing the optimal threshold of Rac1 expression within the diagnostic cohort, a segregation was performed on the 198 patients afflicted with gastric cancer, resulting in the formation of two distinct groups: one characterized by elevated Rac1 expression, and the other marked by diminished Rac1 expression. Of the total sample size, a majority of 63.6% (126 out of 198) exhibited a diminished level of Rac1 expression, while the remaining 36.4% (72 out of 198) displayed an elevated Rac1 expression. **Table 1** showcases the fundamental attributes of these distinct groups. The findings demonstrated a notable association between the expression of Rac1 and various factors, including tumor size (*P*=0.013), location (*P*=0.031), N stage (*P*=0.002), TNM stage (*P*=0.035), rate of positive lymph nodes (*P*<0.001), as well as survival status (*P*<0.001). The prevalence of Rac1 in TNM stage III-IV specimens was determined to be 41.9%, a statistically significant increase compared to the prevalence in TNM stage I-II specimens (27.0%). Nevertheless, no significant associations were observed between Rac1 expression and age, gender, pathological type, pathological grade, lymphatic invasion, neural invasion, T stage, or M stage when employing a significance level of *P*<0.05 (**Table 1**).

Patients exhibiting elevated levels of Rac1 expression demonstrated markedly lower overall survival rates compared to patients with negative Rac1 expression (*P*<0.0001, The log-rank test, *χ^2^ = *27.060). Patient survival according to Rac1 expression over time is illustrated in **Figure 3B**. The predictive prognostic performance of Rac1 was evaluated using ROC analysis, which yielded an AUC of 0.708 (95% CI: 0.636-0.780, sensitivity: 50.9%, specificity: 79.3%) (**Figure 3C**). We additionally crafted ROC curves and computed AUC scores to investigate the potential of Rac1 as a biomarker for prognosticating lymph node metastasis, yielding an AUC value of 0.638. (95% CI: 0.551-0.725, sensitivity: 40.4%, specificity: 74.5%) (**Figure 3D**).

### 3.5 The relationship between ITGB6 and Rac1 expression in gastric cancer

The elevated expression level of Rac1 was observed to be 53.2% in tissues exhibiting high ITGB6 expression, while it was 25.2% in tissues demonstrating low ITGB6 expression. Spearman correlation analysis unveiled a positive association between Rac1 and ITGB6 expression (*r*=0.285, *P*<0.001, **Table 2**).

**Table 2:** The correlation between the expression of ITGB6 and Rac1 in human gastric tumor tissues (r =0.285, P <0.001).

ITGB6	Rac1		Total
	High	Low	
High	42	37	79
Low	30	89	119
Total	72	126	198

**Figure 4:**
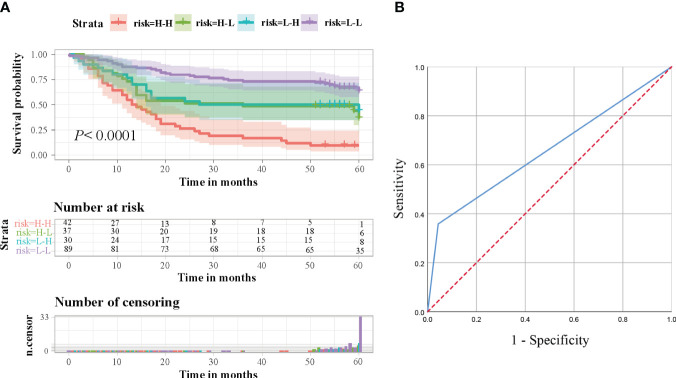
**(A)** Survival analysis was conducted on gastric cancer patients in a retrospective cohort, taking into consideration the combined levels of ITGB6 and Rac1. Based on the expression levels of ITGB6 and Rac1, the patients were classified into four groups, and subsequently, survival rates were calculated and represented using Kaplan-Meier curves. **(B)** In order to predict the prognosis of gastric cancer patients, a Receiver Operating Characteristic (ROC) analysis was performed, utilizing the combined expression levels of ITGB6 and Rac1.

Based on the expression levels of ITGB6 and Rac1, 198 patients were categorized into four distinct groups: group 1 comprised individuals with low levels of both Rac1 and ITGB6 (n=89); group 2 consisted of patients with low Rac1 but high ITGB6 levels (n=37); group 3 included individuals with high Rac1 but low ITGB6 levels (n=30); and group 4 comprised patients with high levels of both Rac1 and ITGB6 (n=42). It was observed that patients in the high Rac1/high integrin αvβ6 group exhibited a significantly poorer overall survival rate compared to the other groups(*P*<0.0001, The log-rank test, *χ^2^ = *35.712). The chart in **Figure 4A** exhibits the long-term survival outcomes of individuals who possess Rac1 expressing ITGB6. Furthermore, the area under the receiver operating characteristic (ROC) curve (AUC) for the amalgamated two biomarkers escalated to 0.658 (95% *CI*, 0.582-0.733), accompanied by estimations of sensitivity and specificity amounting to 35.8% and 95.7%, correspondingly (**Figure 4B**).

### 3.6 Univariate and multivariate analysis for prognosis of patients with gastric cancer

In this investigation, we conducted univariate and multivariate analyses of the data to ascertain the prognostic significance of ITGB6 and Rac1 expression through the utilization of Cox proportional hazards regression models. The age surpasses the designated threshold (*P*=0.001), Pathological grading (*P*=0.016), tumor size (*P*<0.001), Lymphatic invasion (*P*=0.001), T stage (*P*=0.003), N stage (*P*<0.001), M stage (*P*<0.001), clinical stage (*P*<0.001), high αvβ6 expression (*P*<0.001) and high Rac1 expression (P<0.001) were identified as determinants indicating an unfavorable prognosis in the univariate analysis (as indicated in **Table 3**). Then a significance level of *P*<0.10 was utilized as a variable in the multivariate analysis. Through this analysis, it was discovered that elevated αvβ6 expression and heightened Rac1 expression served as unfavorable independent prognostic factors (relative risk (*RR*): 2.212 and 2.073; *P*<0.001 and *P*=0.001, respectively). Notably, age, tumor size, and TNM stage also emerged as independent prognostic factors (*RR*: 2.977, 2.553, and 1.760; *P*<0.001, *P*=0.003, and *P*=0.035, respectively) (As presented in **Table 3**).

**Table 3:** Univariate and multivariate analyses applying the Cox proportional hazard model to patients diagnosed with gastric cancer (Retrospective cohort).

	Univariate analysis			Multivariate analysis		
Variable	HR	95%CI	*P-*value	HR	95%CI	*P*-value
Gender	0.946	0.631-1.418	0.787			
Age(years)	2.135	1.376-3.313	0.001	2.977	1.834-4.833	<0.001
Pathological type Adenocarcinoma Adenocarcinoma, mucinous Carcinoma,Signet Ring Cell Carcinoma, Undifferentiated	Reference 1.151 1.396 3.515	- 0.593-2.233 0.877-2.222 0.857-14.424	0.198 - 0.678 0.159 0.081			
Pathological grading	1.761	1.109-2.795	0.016	1.609	0.968-2.673	0.067
tumor size 1-6.5 7-9 9.5-19	Reference 1.683 3.118	- 1.090-2.600 1.867-5.205	<0.001 - 0.019 <0.001	Reference 1.526 2.553	- 0.970-2.402 1.469-4.435	0.003 - 0.068 0.001
location upper third middle third lower third	Reference 1.258 1.100	- 0.793-1.994 0.678-1.784	0.615 - 0.330 0.699	Reference 0.867 1.100	- 0.531-1.415 0.668-1.813	0.627 - 0.569 0.707
Lymphatic invasion	1.907	1.298-2.803	0.001	1.492	0.918-2.424	0.106
Nerve invasion	1.494	0.989-2.256	0.057	0.942	0.586-1.514	0.804
T stage	3.012	1.464-6.196	0.003			
N stage N0 N1 N2 N3	Reference 2.602 3.385 6.669	- 1.169-5.792 1.648-6.951 3.378-13.169	<0.001 - 0.019 0.001 <0.001			
M stage	2.996	2.474-19.778	<0.001			
TNM stage	2.921	1.850-4.613	<0.001	1.760	1.042-2.974	0.035
ITGB6	2.847	1.930-4.199	<0.001	2.212	1.430-3.423	<0.001
Rac1	2.650	1.804-3.894	<0.001	2.073	1.346-3.191	0.001

HR, hazard ratio; CI, confidence interval.

### 3.7 Establish and verify the Nomogram model of ITGB6 and Rac1

**Figure 5:**
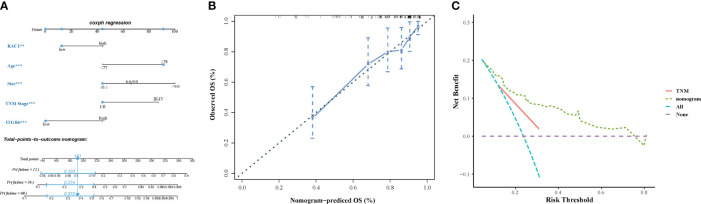
**(A)** A prognostic nomogram displaying the 1-year, 2-year, 3-year, and 5-year overall survival rates of 198 individuals diagnosed with gastric cancer. **(B)** The calibration curves assessing the predictive accuracy of the nomograms in estimating the overall survival rates of gastric cancer patients. **(C)** The decision curve analysis (DCA) comparing the performance of the nomograms and the TNM system in predicting the overall survival rates of gastric cancer patients.

The final model for constructing a prognostic nomogram predicting overall survival (**Figure 5A**) incorporated age, tumor size, TNM stage, ITGB6, and Rac1 expression based on the aforementioned findings. **Table 4** shows the nomogram prognostic factor scores. In order to assess the discernment of the Nomogram, we employed the C-index. The prognostication model demonstrated remarkable precision, exhibiting a C-index of 0.751(95% CI: 0.704-0.798). Meticulously developed calibration curves were implemented to evaluate the congruity between the prognosticated probabilities derived from the nomogram and the veritable observed survival rates. The calibration plot of postoperative OS showed that the predicted combined expression of ITGB6 and Rac1 based on nomogram was basically consistent with the actual observation (**Figure 5B**). In order to assess the practical value of the OS nomogram, the methodology of decision curve analysis (DCA) was employed to quantify the overall advantage at various threshold probabilities. The clinical applicability and advantages of the OS nomogram were contrasted with those of the TNM using DCA. The graphical representation of DCA demonstrated that the nomogram exhibited superior prediction and clinical relevance compared to TNM (**Figure 5C**).

**Table 4:** Numerous prognostic factors are incorporated within the OS and CSS nomograms.

Characteristic	OS nomogram
Age (years)	
<77	44
≥80	91
TumorSize	
1-6.5	44
7-9	66
9.5-19	100
TNM	
I-II	44
III-IV	87
ITGB6	
High	44
Low	0
Rac1	
High	44
Low	12

### 3.8 Functional validation in cellular models

**Figure 6:**
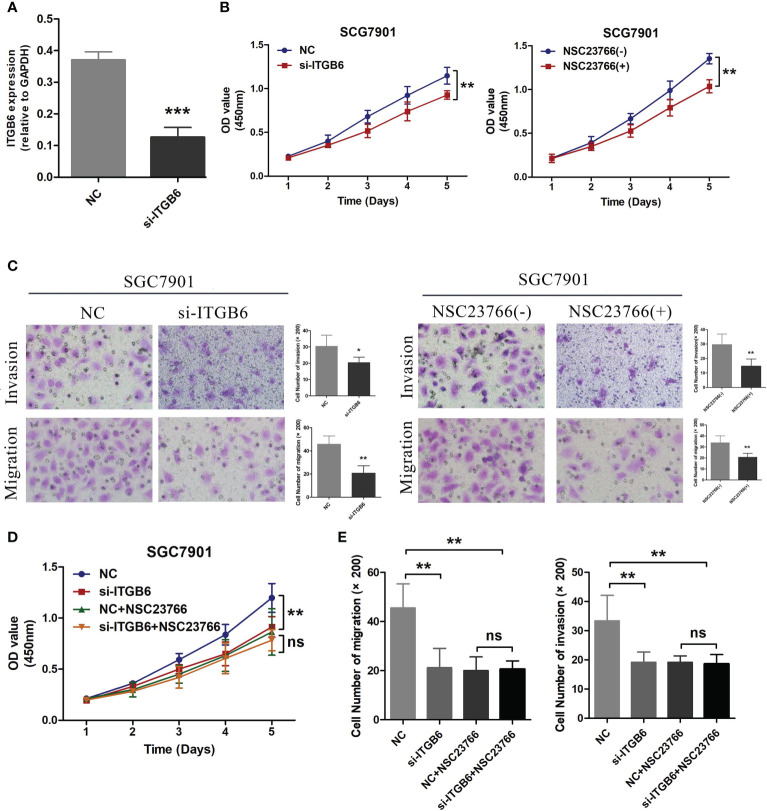
**(A)** Transfection of ITGB6 siRNA was conducted in the SGC7901 gastric cancer cell line, and the transfection efficiency was evaluated using RT-PCR. **(B)** After inhibiting the expression of ITGB6 in 7901 cells and treating them with the Rac1 activity inhibitor NSC23766, the cell viability was assessed using the CCK8 assays. **(C)** After interfering with ITGB6 expression in 7901 cells and treating them with the Rac1 activity inhibitor NSC23766, Transwell migration and invasion assays were performed to assess the changes in cell migration and invasion abilities. **(D)** Transfection of NC and ITGB6 siRNA was performed separately in 7901 cells, followed by treatment with NSC23766. Cell viability was evaluated using the CCK8 assay. **(E)** In the experiment, we performed co-transfection of NC and ITGB6 siRNA in 7901 cells, accompanied by NSC23766 treatment. The migration and invasion abilities of the cells were assessed through Transwell migration and invasion assays. *P<0.05; **P<0.001; ***P<0.0001

The gastric cancer cell line 7901 was cultured and the expression of ITGB6 was downregulated by transfection with ITGB6 interfering RNA. The siRNA effect was confirmed by RT-PCR (**Figure 6A**). Subsequently, CCK8 experiments were used to investigate changes in cell activity after ITGB6 interference, and it was found that downregulation of ITGB6 can significantly inhibit gastric cancer cell activity. In addition, the Rac1 activity inhibitor NSC23766 had a similar effect to si-ITGB6 (**Figure 6B**). Transwell migration and invasion experiments (with Matri-gel) were conducted to explore the effects of si-ITGB6 and Rac1 inhibitor on the migration and invasion ability of gastric cancer cells. It was found that downregulation of ITGB6 expression and Rac1 activity inhibition could significantly inhibit the migration and invasion ability of gastric cancer cells (**Figure 6C**). To further validate this effect, we treated gastric cancer cell lines transfected with NC, si-ITGB6, and NSC23766. Simultaneously, experimental results showed that after inhibiting Rac1 activity, the role of ITGB6 in gastric cancer cell activity, migration, and invasion disappeared, suggesting that ITGB6 might play a role in the proliferation, migration, and invasion of gastric cancer cells through Rac1 (**Figures 6D, E**).

## 4 Discussion

Gastric cancer has high morbidity and mortality worldwide (21). Although there have been notable advancements in the screening and management of gastric cancer in recent times, the clinical outcomes remain inadequate. Gaining insight into the molecular mechanisms underlying tumorigenesis is imperative in order to enhance the prognosis of afflicted individuals.

ITGB6 can be found in various epithelial tumors and plays a crucial role in the invasion and spread of cancer (22). In order to delve deeper into the expression of ITGB6 in cancer, we conducted immunostaining analysis on a total of 198 patient samples. We have observed that ITGB6 exhibits higher expression in gastric tumor tissues and lymph node metastasis, compared to adjacent non-cancerous tissues and lymph node metastasis negative. Recent studies have identified it as an independent predictor of poorer prognosis in aggressive forms of colorectal and gastric cancers, a finding that has also been confirmed in our own study using the Cox regression model (23, 24). Similar to Bates’s study (24), additionally, our findings indicated a significant correlation between the expression of ITGB6 and both the clinical stage and invasion depth of tumors. These observed associations are consistent with previous research and can be plausibly elucidated. ITGB6 possesses the ability to guide the growth factor-triggered activation of ERK towards subsequent cytoplasmic objectives, thereby contributing to the control of cellular proliferation, programmed cell death, and the rearrangement of the cytoskeleton. Additionally, it aids in facilitating cellular migration by regulating the release of MMP-9 (25–27). Moreover, patients with elevated ITGB6 expression exhibit poorer overall survival.

In recent decades, research has presented compelling evidence regarding the pivotal involvement of Rac1 in the advancement and proliferation of cancer cells (28–30). Rac1 has been demonstrated to enhance cellular proliferation by activating downstream signaling pathways that stimulate the progression of the cell cycle and hinder cellular demise (31). And this point has also been corroborated through immunohistochemistry, revealing that Rac1 is highly expressed in gastric tumor tissue and lymph node metastasis, serving as an independent prognostic factor for gastric cancer patients and leading to poorer overall survival. Due to its crucial role in tumor development, Rac1 has become a standard for tumor stratification and a promising therapeutic target (32, 33).

Multiple investigations have indicated that the activation of Rac1 is prompted through its interaction with ITGB6, one of its downstream effectors, thereby augmenting the migratory and invasive capabilities of tumor cells. In order to substantiate the significance of Rac1 in ITGB6-dependent invasion, Paul H. Weinreb and his associates employed RNA interference techniques to suppress Rac1 expression. Their findings verified that Rac1 orchestrates the ITGB6-dependent invasion process. Consistent with these findings, our data indicate that the activation of Rac1 by ITGB6 promotes the invasion and metastasis of gastric cancer cells. The expression levels of ITGB6 and Rac1 are positively correlated, and the group with high expression of both ITGB6 and Rac1 exhibits poorer overall survival. Additionally, our experiments using cell transfection and Transwell invasion/migration assay demonstrate that si-ITGB6 can inhibit tumor invasion and metastatic abilities. And vice versa, inhibiting the activity of Rac1 results in decreased proliferation, invasion, and metastasis capabilities of gastric cancer cells expressing ITGB6. Importantly, our study showed that the expression levels of ITGB6 and Rac1 are associated with the unfavorable prognosis of gastric cancer patients. Building upon the aforementioned findings, a nomogram forecasting overall survival in gastric cancer patients was devised, relying on the expression of ITGB6 and Rac1. This nomogram demonstrates a commendable predictive efficacy. Furthermore, studies have revealed that Rac1 intricately participates in diverse tumorigenic signaling pathways, encompassing the JNK/SAPK and ERK/MAPK cascades (34, 35). In a manner contingent upon COX-2, ITGB6 has exhibited the ability to elicit the activation of Rac1, whereby the regulation of said activation may be influenced by the genetic composition responsible for encoding the epidermal growth factor receptor pathway substrate 8 (Eps8) (36).

Nonetheless, there are a few potential limitations in this study that require attention. Primarily, the sample size utilized in this study was relatively modest, thereby necessitating the inclusion of studies with more substantial sample sizes to validate the conclusiveness of the outcomes. Secondly, in our current investigation, we ascertained that the confluence of ITGB6 positivity and elevated Rac1 expression portends an unfavorable prognosis. However, it is worth noting that we employed an IHC staining technique, which could potentially introduce interobserver variability and influence the obtained results. To mitigate this concern, we engaged the expertise of two seasoned pathologists during the scoring process. Nevertheless, further research is still warranted to elucidate its underlying molecular mechanism.

## 5 Conclusion

To conclude, our study findings indicate that the levels of ITGB6 and Rac1 are heightened in gastric carcinoma and exhibit a correlation, thereby linking them to tumor advancement and unfavorable prognosis among gastric cancer patients. These findings have important implications for the development of potential therapeutic interventions.

## Data availability statement

The data used to support the findings of this study may be released upon application to the Department of Gastrointestinal Surgery, The Affiliated Hospital of Qingdao University, who can be contacted at shougencao@qdu.edu.cn.

## Ethics statement

This study followed the principles outlined in the Declaration of Helsinki and was granted approval by the Ethics Committee of the Affiliated Hospital of Qingdao University (No.QDFY27398).

## Author contributions

JY: Conceptualization, Data curation, Formal analysis, Investigation, Methodology, Project administration, Resources, Software, Validation, Visualization, Writing – original draft. WJ: Data curation, Formal analysis, Investigation, Methodology, Project administration, Resources, Software, Validation, Visualization, Writing – original draft. QL: Data curation, Formal analysis, Software, Writing – original draft. AY: Data curation, Formal analysis, Software, Writing – original draft. ZJ: Data curation, Formal analysis, Methodology, Writing – original draft. YS: Conceptualization, Data curation, Methodology, Software, Supervision, Writing – original draft, Writing – review & editing. ZL: Resources, Supervision, Writing – review & editing. SC: Conceptualization, Methodology, Supervision, Writing – review & editing.
